# Supplementary material for: Transcriptomic Insights into Mechanisms of Early Seed Maturation in the Garden Pea (Pisum sativum L.)
Source: Cells. 2020 Mar 23;9(3):779. doi: 10.3390/cells9030779 (PMC7140803; doi:10.3390/cells9030779)
Supplement: Supplementary file 1 [file cells-09-00779-s001.zip › Supplementary materials/FileS2.zip!/FileS1/Summary Sheets - Modules/K-Means Cluster/Report.pdf]

K−Means Cluster

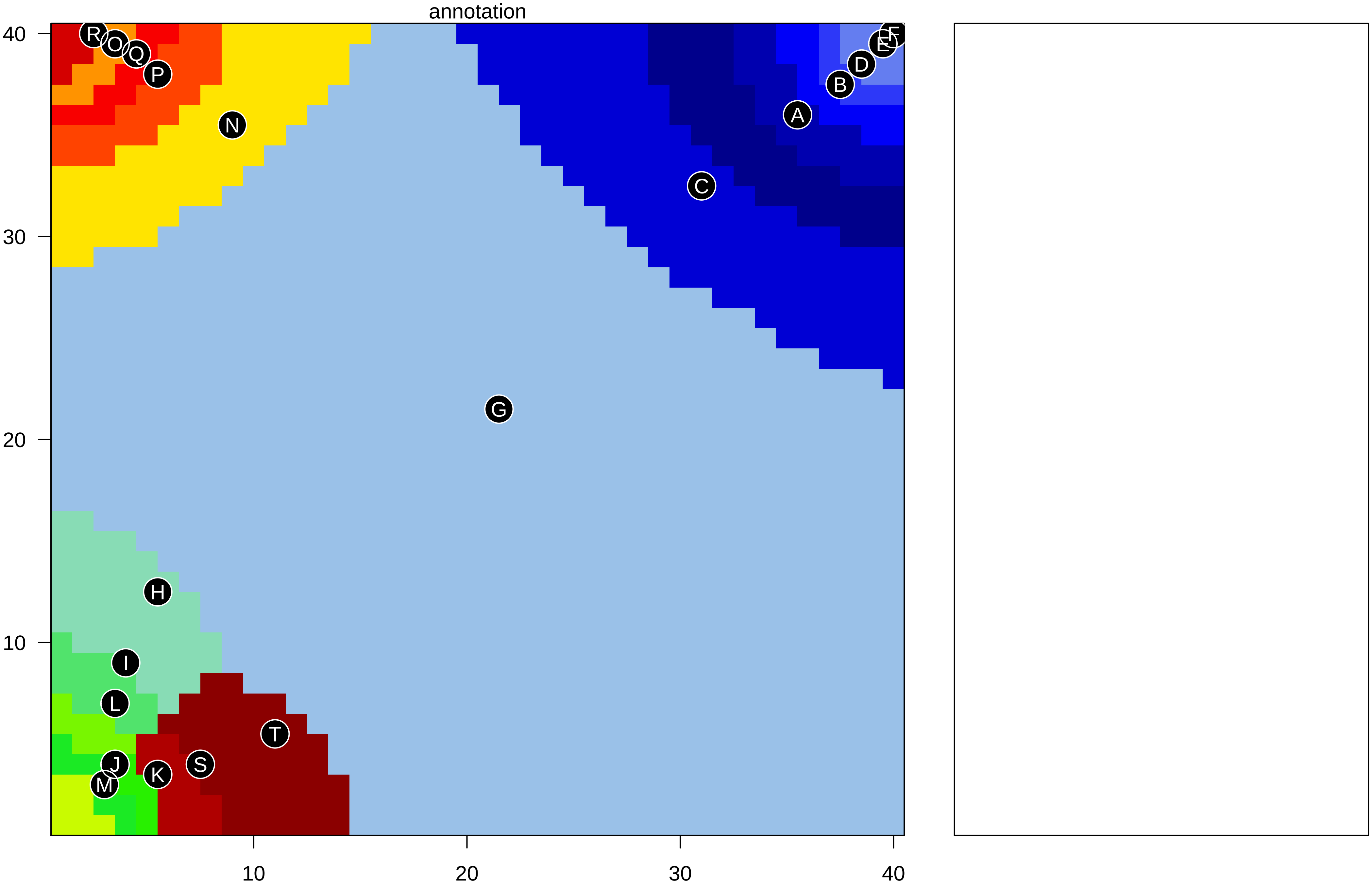

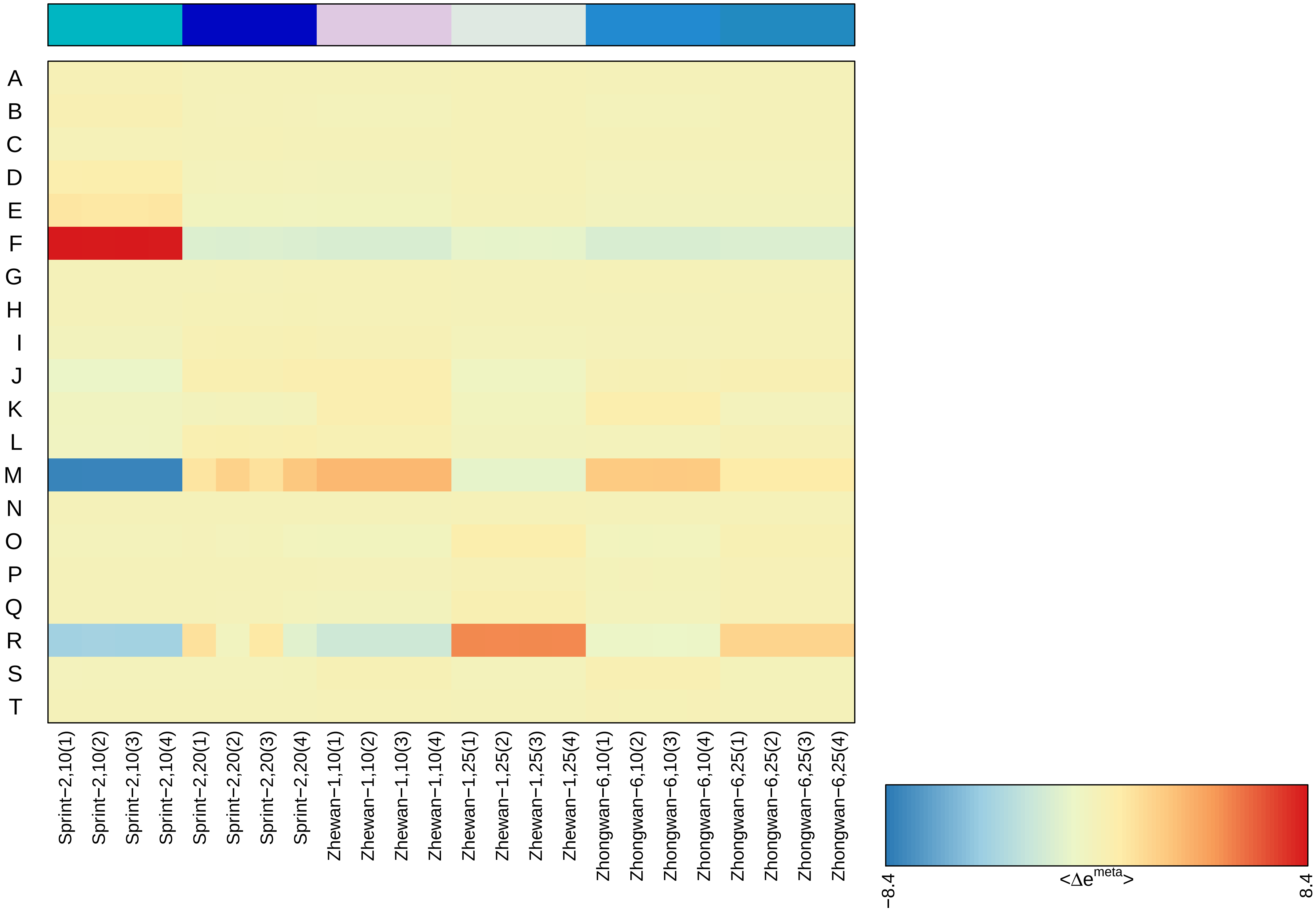
A
B
C
D
E
F
G
H
I
J
K
L
M
N
O
P
Q
R
S
T
Sprint−2,10(1)
Sprint−2,10(2)
Sprint−2,10(3)
Sprint−2,10(4)
Sprint−2,20(1)
Sprint−2,20(2)
Sprint−2,20(3)
Sprint−2,20(4)
Zhewan−1,10(1)
Zhewan−1,10(2)
Zhewan−1,10(3)
Zhewan−1,10(4)
Zhewan−1,25(1)
Zhewan−1,25(2)
Zhewan−1,25(3)
Zhewan−1,25(4)
Zhongwan−6,10(1)
Zhongwan−6,10(2)
Zhongwan−6,10(3)
Zhongwan−6,10(4)
Zhongwan−6,25(1)
Zhongwan−6,25(2)
Zhongwan−6,25(3)
Zhongwan−6,25(4)
−8.4
<Δe^meta>
8.4

K−Means Cluster

Spot Summary: A

metagenes = 48
genes = 345

<r> metagenes = 0.92
<r> genes = 0.74
beta: r2= 0.14 / log p= −1.15

samples with spot = 0 (0 %)

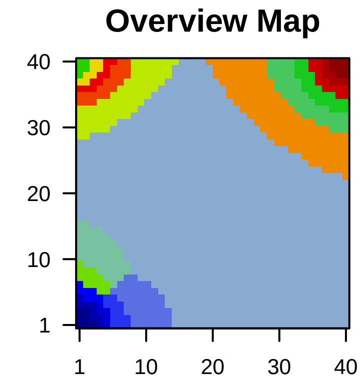

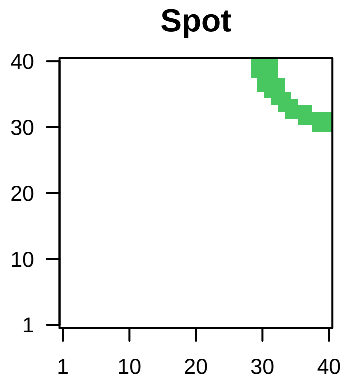

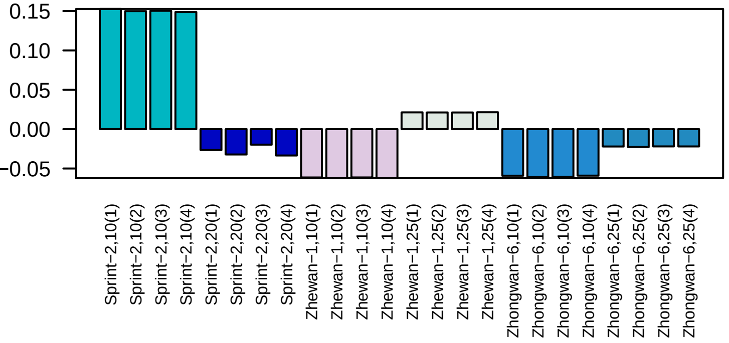

Spot Genelist

Rank	ID	max e	min e	r	Symbol	Description
1	TRINITY_DN2	0.41	−0.15	0.45		
2	TRINITY_DN5	0.35	−0.06	0.9		
3	TRINITY_DN4	0.34	−0.14	0.82		
4	TRINITY_DN5	0.33	−0.05	0.9		
5	TRINITY_DN3	0.32	−0.05	0.9		
6	TRINITY_DN2	0.31	−0.12	0.75		
7	TRINITY_DN1	0.3	−0.21	0.44		
8	TRINITY_DN7	0.3	−0.05	0.91		
9	TRINITY_DN1	0.3	−0.05	0.91		
10	TRINITY_DN1	0.3	−0.14	0.86		
11	TRINITY_DN5	0.3	−0.08	0.94		
12	TRINITY_DN2	0.3	−0.28	0.69		
13	TRINITY_DN3	0.29	−0.16	0.89		
14	TRINITY_DN4	0.29	−0.12	0.95		
15	TRINITY_DN4	0.29	−0.08	0.88		
16	TRINITY_DN4	0.29	−0.07	0.86		
17	TRINITY_DN3	0.29	−0.22	0.71		
18	TRINITY_DN1	0.29	−0.05	0.93		
19	TRINITY_DN1	0.28	−0.08	0.9		
20	TRINITY_DN9	0.28	−0.12	0.83		

K−Means Cluster

Spot Summary: B

metagenes = 24
genes = 190

<r> metagenes = 0.96
<r> genes = 0.77
beta: r2= 0.63 / log p= −5.42

samples with spot = 0 (0 %)

Spot Genelist

Rank	ID	max e	min e	r	Symbol	Description
1	TRINITY_DN5	0.7	−0.09	0.88		
2	TRINITY_DN4	0.69	−0.09	0.86		
3	TRINITY_DN5	0.66	−0.11	0.91		
4	TRINITY_DN4	0.65	−0.35	0.94		
5	TRINITY_DN5	0.62	−0.12	0.77		
6	TRINITY_DN2	0.62	−0.11	0.93		
7	TRINITY_DN6	0.61	−0.35	0.87		
8	TRINITY_DN2	0.6	−0.21	0.93		
9	TRINITY_DN3	0.6	−0.13	0.94		
10	TRINITY_DN3	0.59	−0.09	0.9		
11	TRINITY_DN1	0.59	−0.26	0.7		
12	TRINITY_DN8	0.59	−0.16	0.95		
13	TRINITY_DN5	0.59	−0.18	0.98		
14	comp19684_c	0.59	−0.23	0.9		
15	TRINITY_DN3	0.58	−0.11	0.9		
16	TRINITY_DN9	0.58	−0.12	0.94		
17	TRINITY_DN2	0.57	−0.19	0.95		
18	TRINITY_DN1	0.57	−0.16	0.95		
19	TRINITY_DN1	0.57	−0.23	0.82		
20	TRINITY_DN5	0.56	−0.1	0.92		

Overview Map

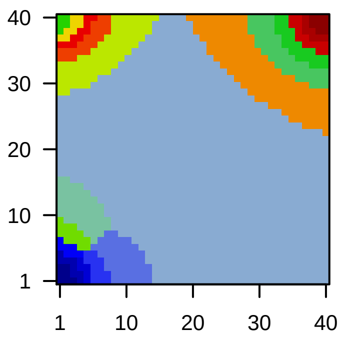

Spot

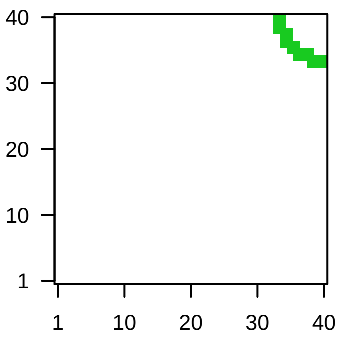

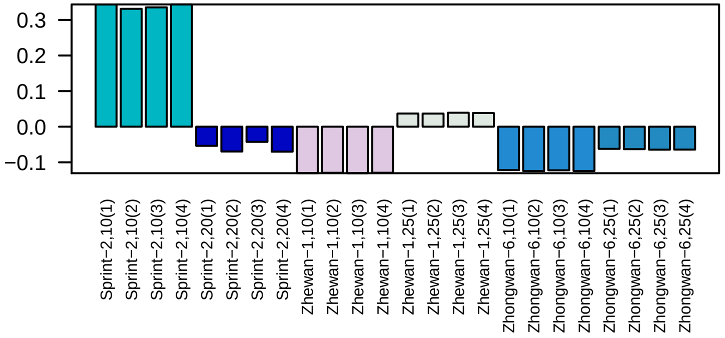

K−Means Cluster

Spot Summary: C

metagenes = 141
genes = 1430

<r> metagenes = 0.85

beta: r2= 0.02 / log p= −0.27

samples with spot = 0 (0 %)

Overview Map

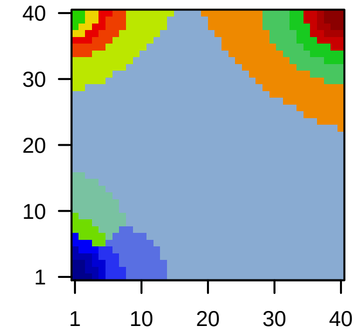

Spot

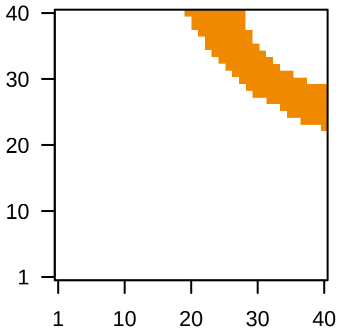

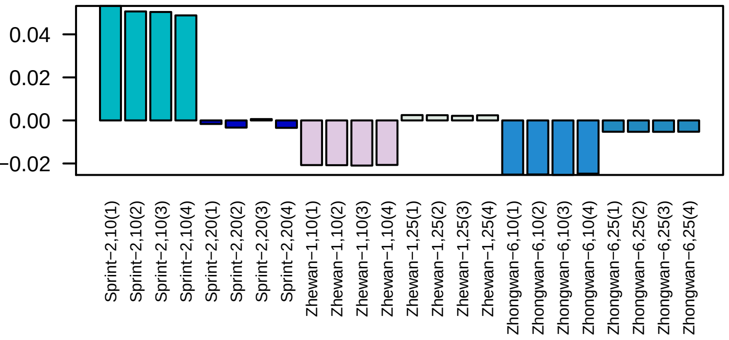

Spot Genelist

Rank	ID	max e	min e	r	Symbol	Description
1	TRINITY_DN3	0.31	−0.24	0.12		
2	comp33880_c	0.28	−0.17	0.63		
3	TRINITY_DN1	0.25	−0.13	0.54		
4	TRINITY_DN2	0.25	−0.2	0.53		
5	TRINITY_DN8	0.23	−0.17	0.38		
6	TRINITY_DN1	0.23	−0.16	0.66		
7	TRINITY_DN3	0.22	−0.21	0.48		
8	TRINITY_DN1	0.22	−0.2	0.51		
9	TRINITY_DN6	0.22	−0.02	0.79		
10	TRINITY_DN1	0.2	−0.02	0.75		
11	TRINITY_DN4	0.19	−0.19	0.71		
12	TRINITY_DN4	0.19	−0.08	0.63		
13	TRINITY_DN5	0.18	−0.2	0.38		
14	TRINITY_DN2	0.18	−0.13	0.53		
15	comp9719_c0	0.16	−0.09	0.75		
16	TRINITY_DN3	0.15	−0.11	0.7		
17	TRINITY_DN1	0.15	−0.06	0.88		
18	comp35381_c	0.15	−0.05	0.72		
19	TRINITY_DN9	0.15	−0.16	0.32		
20	comp13822_c	0.14	−0.07	0.92		

K−Means Cluster

Spot Summary: D

metagenes = 13
genes = 73

<r> metagenes = 0.99
<r> genes = 0.82
beta: r2= 2.53 / log p= −Inf

samples with spot = 0 (0 %)

Spot Genelist

Rank	ID	max e	min e	r	Symbol	Description
1	TRINITY_DN3	2.23	−1.5	0.28		
2	TRINITY_DN8	1.47	−0.62	0.94		
3	TRINITY_DN5	1.44	−0.42	0.86		
4	TRINITY_DN7	1.32	−0.22	0.95		
5	TRINITY_DN6	1.29	−0.49	0.68		
6	TRINITY_DN2	1.18	−0.27	0.97		
7	TRINITY_DN4	1.18	−0.2	0.92		
8	TRINITY_DN1	1.15	−0.42	0.97		
9	TRINITY_DN7	1.13	−0.29	0.97		
10	TRINITY_DN7	1.13	−0.24	0.95		
11	TRINITY_DN2	1.09	−0.72	0.89		
12	TRINITY_DN4	1.08	−0.2	0.96		
13	TRINITY_DN3	1.08	−0.19	0.94		
14	TRINITY_DN2	1.08	−0.23	0.98		
15	TRINITY_DN2	1.06	−0.18	0.94		
16	TRINITY_DN2	1.04	−0.63	0.83		
17	TRINITY_DN4	1.04	−0.18	0.95		
18	TRINITY_DN8	1.04	−0.17	0.95		
19	TRINITY_DN1	1.03	−0.51	0.91		
20	TRINITY_DN6	1	−0.27	0.96		

Overview Map

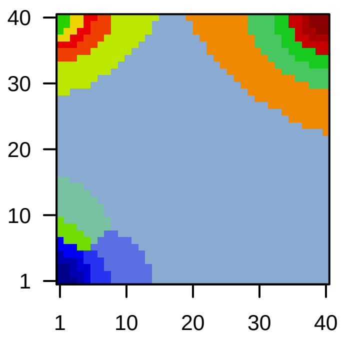

Spot

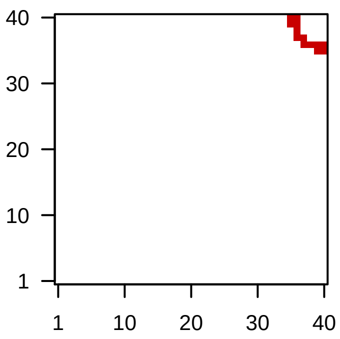

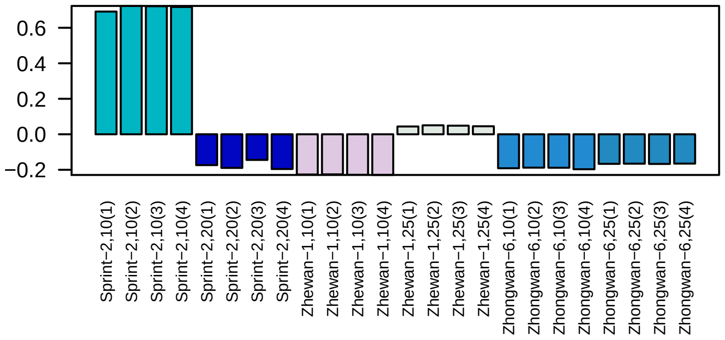

K−Means Cluster

Spot Summary: E

metagenes = 7
genes = 20

<r> metagenes = 1
<r> genes = 0.88
beta: r2= 7.22 / log p= −Inf

samples with spot = 0 (0 %)

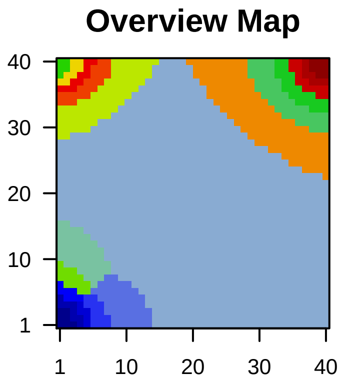

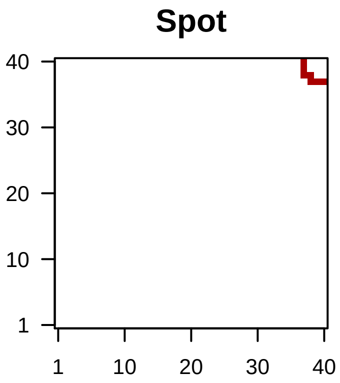

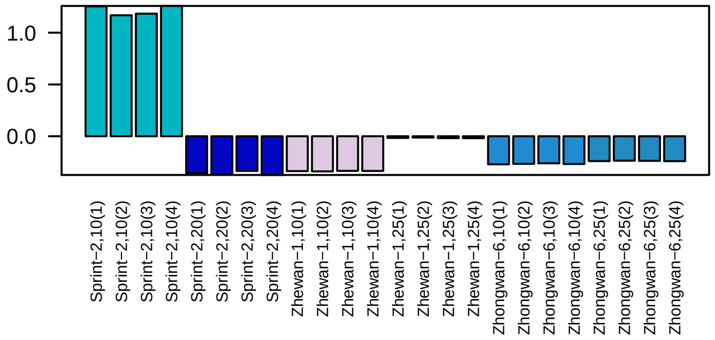

Spot Genelist

Rank	ID	max e	min e	r	Symbol	Description
1	TRINITY_DN6	1.99	−0.97	0.85		
2	TRINITY_DN6	1.99	−0.39	0.95		
3	TRINITY_DN2	1.87	−0.31	0.97		
4	TRINITY_DN2	1.86	−0.61	0.96		
5	TRINITY_DN1	1.81	−0.28	0.96		
6	TRINITY_DN3	1.8	−0.3	0.96		
7	TRINITY_DN5	1.56	−0.91	0.82		
8	TRINITY_DN8	1.54	−0.3	0.98		
9	TRINITY_DN9	1.49	−0.33	0.94		
10	TRINITY_DN5	1.45	−0.58	0.96		
11	TRINITY_DN3	1.44	−0.7	0.95		
12	TRINITY_DN4	1.43	−0.72	0.76		
13	TRINITY_DN4	1.43	−0.48	0.91		
14	TRINITY_DN4	1.38	−0.27	0.97		
15	TRINITY_DN2	1.38	−0.48	0.94		
16	TRINITY_DN3	1.32	−0.26	0.98		
17	TRINITY_DN4	1.24	−0.3	0.97		
18	TRINITY_DN4	1.23	−0.26	0.98		
19	TRINITY_DN2	1.18	−0.34	0.97		
20	TRINITY_DN5	1.1	−0.34	0.96		

K−Means Cluster

Spot Summary: F

metagenes = 8
genes = 46

<r> metagenes = 1
<r> genes = 0.88
beta: r2= 30.88 / log p= −Inf

samples with spot = 4 (16.7 %)
Sprint−2(10) : 4 (100 %)

Overview Map

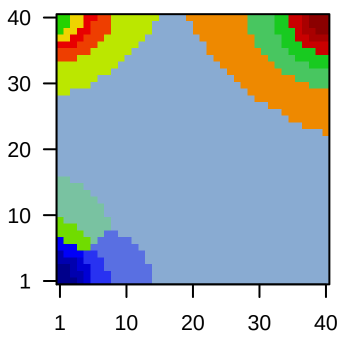

Spot

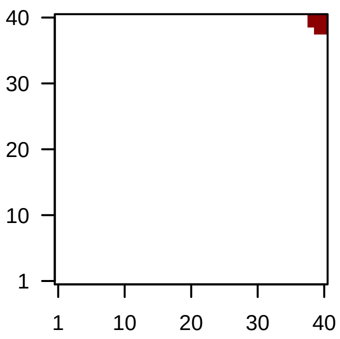

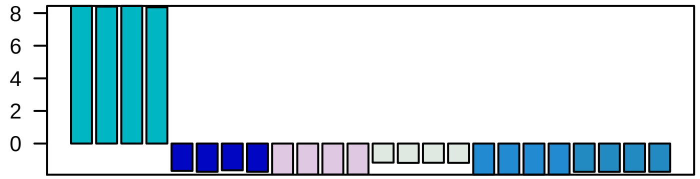

Spot Genelist

Rank	ID	max e	min e	r	Symbol	Description
1	TRINITY_DN7	117.83	−10.22	0.78		
2	TRINITY_DN2	109.03	−18.72	0.93		
3	TRINITY_DN1	36.31	−5.78	0.97		
4	TRINITY_DN8	27.57	−5.74	0.99		
5	TRINITY_DN4	22.28	−3.73	0.99		
6	TRINITY_DN3	14.63	−3.64	1		
7	TRINITY_DN3	13.41	−2.83	0.98		
8	TRINITY_DN8	11.22	−3.35	0.98		
9	TRINITY_DN3	11.09	−2.42	0.98		
10	TRINITY_DN2	10.84	−1.3	0.82		
11	TRINITY_DN5	10.56	−2.04	0.96		
12	TRINITY_DN1	10.49	−2.06	1		
13	TRINITY_DN3	9.42	−1.6	0.98		
14	TRINITY_DN5	9.26	−1.71	0.7		
15	TRINITY_DN1	8.44	−1.61	0.96		
16	TRINITY_DN1	7.68	−1.68	0.96		
17	TRINITY_DN4	7.37	−1.78	0.96		
18	TRINITY_DN1	7.34	−2.04	0.67		
19	TRINITY_DN7	6.84	−1.21	0.99		
20	TRINITY_DN1	6.54	−1.49	0.99		

K−Means Cluster

Spot Summary: G

metagenes = 1093
genes = 23090

<r> metagenes = 0

beta: r2= 0 / log p= −0.03

samples with spot = 0 (0 %)

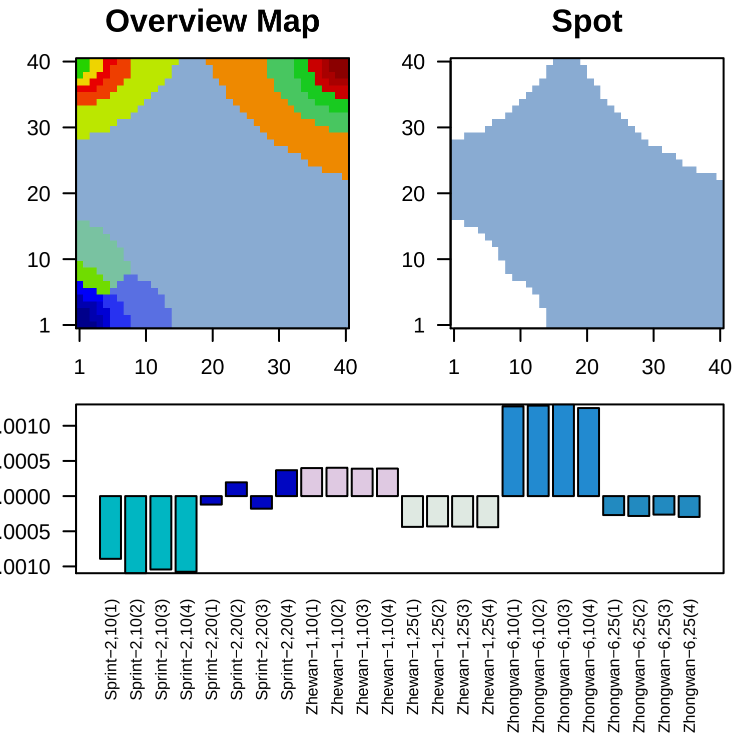

K−Means Cluster

Spot Summary: H

metagenes = 47
genes = 451

<r> metagenes = 0.85
<r> genes = 0.58
beta: r2= 0.06 / log p= −0.59

samples with spot = 0 (0 %)

Spot Genelist

Rank	ID	max e	min e	r	Symbol	Description
1	TRINITY_DN5	0.56	−0.14	0.57		
2	TRINITY_DN2	0.55	−0.16	0.58		
3	TRINITY_DN2	0.45	−0.09	0.8		
4	TRINITY_DN1	0.39	−0.13	0.78		
5	TRINITY_DN3	0.38	−0.2	0.77		
6	TRINITY_DN3	0.35	−0.17	0.46		
7	comp10149_c	0.35	−0.05	0.76		
8	comp39002_c	0.32	−0.1	0.76		
9	TRINITY_DN1	0.31	−0.17	0.68		
10	TRINITY_DN3	0.3	−0.1	0.93		
11	TRINITY_DN4	0.3	−0.1	0.58		
12	comp12834_c	0.3	−0.09	0.57		
13	TRINITY_DN5	0.29	−0.1	0.94		
14	TRINITY_DN4	0.29	−0.17	0.76		
15	TRINITY_DN6	0.29	−0.11	0.59		
16	TRINITY_DN1	0.29	−0.07	0.81		
17	TRINITY_DN2	0.29	−0.16	0.75		
18	TRINITY_DN7	0.28	−0.07	0.9		
19	TRINITY_DN2	0.28	−0.13	0.82		
20	TRINITY_DN1	0.28	−0.1	0.58		

Overview Map

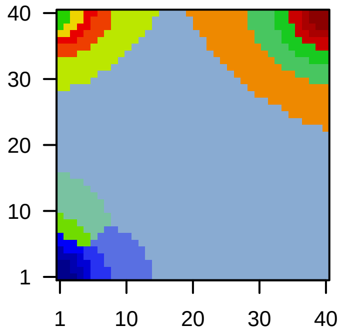

Spot

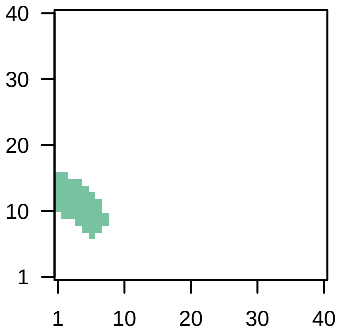

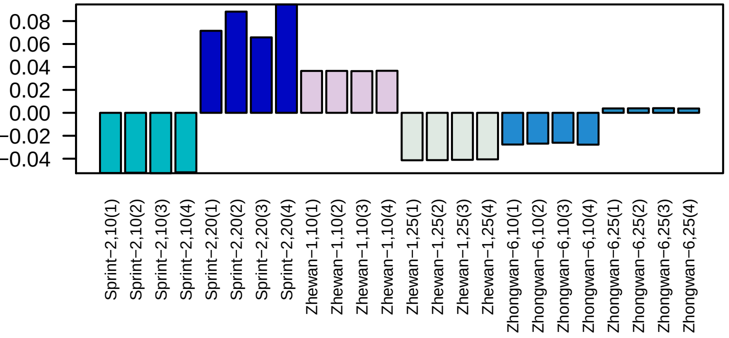

K−Means Cluster

Spot Summary: I

metagenes = 14
genes = 103

<r> metagenes = 0.96
<r> genes = 0.69
beta: r2= 0.52 / log p= −4.17

samples with spot = 0 (0 %)

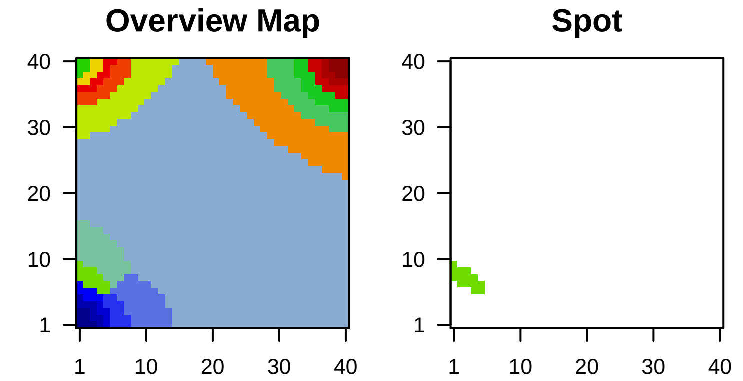

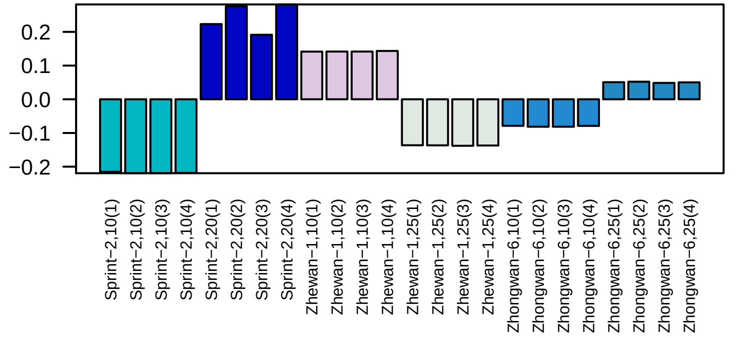

Spot Genelist

Rank	ID	max e	min e	r	Symbol	Description
1	TRINITY_DN1	1.36	−0.32	0.69		
2	TRINITY_DN1	1.24	−0.55	0.65		
3	TRINITY_DN1	1.1	−0.2	0.7		
4	TRINITY_DN4	0.93	−0.2	0.8		
5	TRINITY_DN1	0.69	−0.27	0.96		
6	TRINITY_DN1	0.68	−0.33	0.68		
7	TRINITY_DN2	0.66	−0.22	0.66		
8	TRINITY_DN1	0.65	−0.12	0.74		
9	TRINITY_DN1	0.61	−0.29	0.72		
10	TRINITY_DN3	0.6	−0.22	0.7		
11	TRINITY_DN1	0.57	−0.19	0.83		
12	TRINITY_DN2	0.55	−0.28	0.88		
13	TRINITY_DN2	0.54	−0.29	0.67		
14	TRINITY_DN8	0.53	−0.21	0.44		
15	TRINITY_DN1	0.52	−0.11	0.75		
16	TRINITY_DN2	0.51	−0.21	0.94		
17	TRINITY_DN1	0.51	−0.31	0.89		
18	comp30702_c	0.51	−0.24	0.33		
19	TRINITY_DN3	0.5	−0.29	0.94		
20	TRINITY_DN5	0.49	−0.27	0.88		

K−Means Cluster

Spot Summary: J

metagenes = 8
genes = 51

<r> metagenes = 0.98
<r> genes = 0.7
beta: r2= 6.6 / log p= −Inf

samples with spot = 0 (0 %)

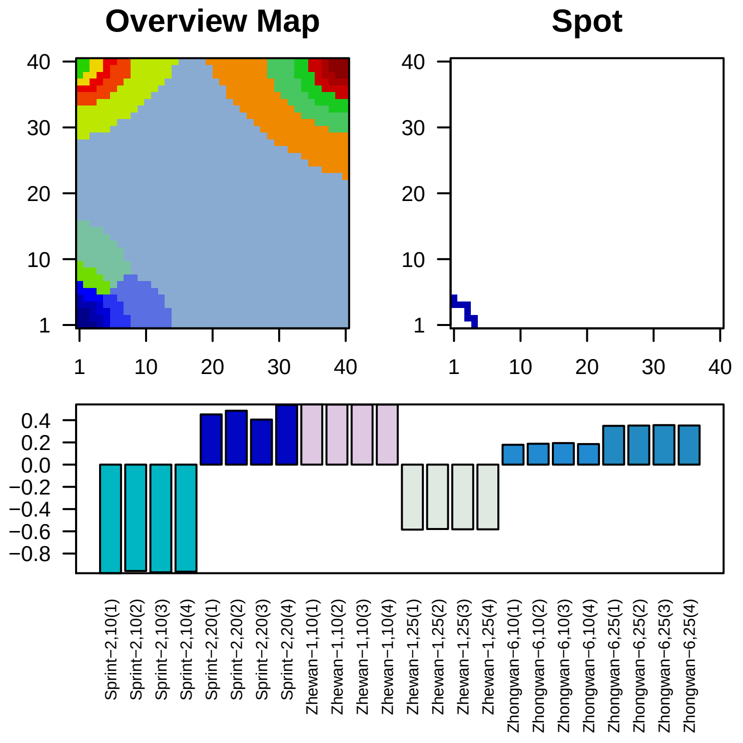

Spot Genelist

Rank	ID	max e	min e	r	Symbol	Description
1	TRINITY_DN2	2.26	−2.02	0.76		
2	TRINITY_DN1	2.07	−1.63	0.7		
3	TRINITY_DN6	2.04	−0.69	0.35		
4	TRINITY_DN1	1.71	−0.67	0.41		
5	TRINITY_DN2	1.55	−0.78	0.75		
6	TRINITY_DN3	1.53	−0.94	0.82		
7	TRINITY_DN3	1.4	−2.05	0.76		
8	TRINITY_DN3	1.39	−1.28	0.86		
9	TRINITY_DN1	1.36	−0.81	0.71		
10	TRINITY_DN5	1.32	−0.78	0.57		
11	TRINITY_DN2	1.27	−1.32	0.87		
12	TRINITY_DN3	1.22	−1.58	0.82		
13	TRINITY_DN2	1.22	−1.35	0.95		
14	TRINITY_DN3	1.16	−1.06	0.92		
15	TRINITY_DN1	1.16	−0.86	0.78		
16	TRINITY_DN2	1.12	−0.84	0.55		
17	TRINITY_DN8	1.04	−1.32	0.89		
18	TRINITY_DN3	1.04	−0.75	0.8		
19	TRINITY_DN2	0.98	−0.7	0.86		
20	TRINITY_DN1	0.92	−0.89	0.96		

K−Means Cluster

Spot Summary: K

metagenes = 5
genes = 26

<r> metagenes = 0.98
<r> genes = 0.86
beta: r2= 2.71 / log p= −Inf

samples with spot = 0 (0 %)

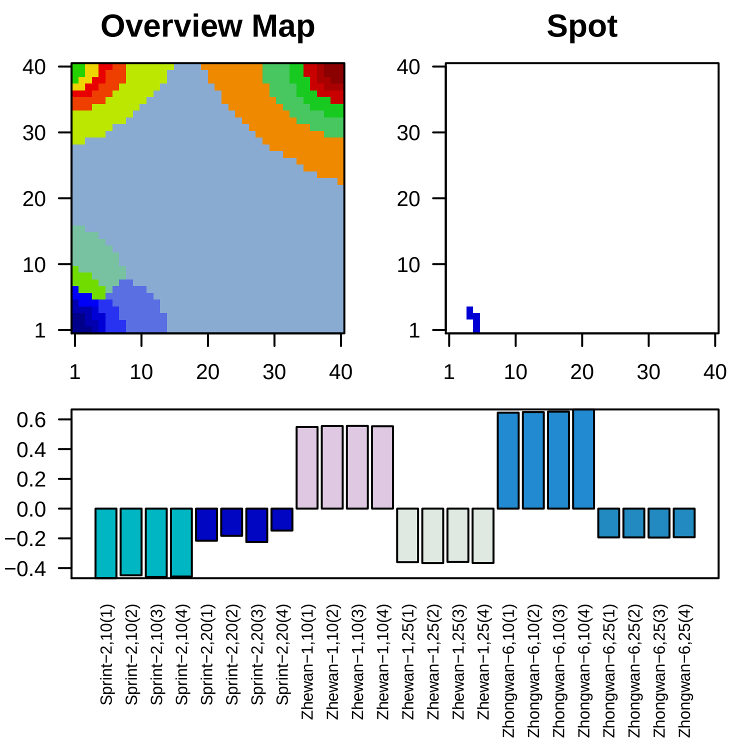

Spot Genelist

Rank	ID	max e	min e	r	Symbol	Description
1	TRINITY_DN2	1.57	−0.54	0.74		
2	comp18180_c	1.54	−0.53	0.91		
3	TRINITY_DN5	1.22	−0.45	0.95		
4	comp14844_c	1.17	−0.6	0.93		
5	TRINITY_DN1	1.08	−0.66	0.9		
6	TRINITY_DN1	1.04	−0.41	0.97		
7	TRINITY_DN1	0.9	−0.42	0.98		
8	TRINITY_DN3	0.87	−0.42	0.98		
9	TRINITY_DN6	0.86	−0.65	0.97		
10	TRINITY_DN9	0.85	−0.36	0.97		
11	TRINITY_DN3	0.77	−0.43	0.99		
12	TRINITY_DN1	0.71	−0.53	0.93		
13	TRINITY_DN4	0.71	−0.43	0.99		
14	TRINITY_DN2	0.7	−0.39	0.99		
15	TRINITY_DN4	0.68	−0.34	0.97		
16	TRINITY_DN3	0.68	−0.41	0.99		
17	TRINITY_DN2	0.67	−0.57	0.9		
18	TRINITY_DN8	0.63	−0.35	0.86		
19	TRINITY_DN1	0.61	−0.33	0.96		
20	TRINITY_DN2	0.56	−0.44	0.99		

K−Means Cluster

Spot Summary: L

metagenes = 7
genes = 43

<r> metagenes = 0.98
<r> genes = 0.75
beta: r2= 1.92 / log p= −Inf

samples with spot = 0 (0 %)

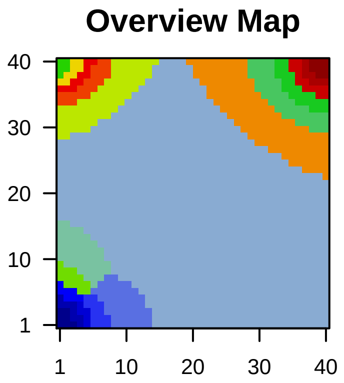

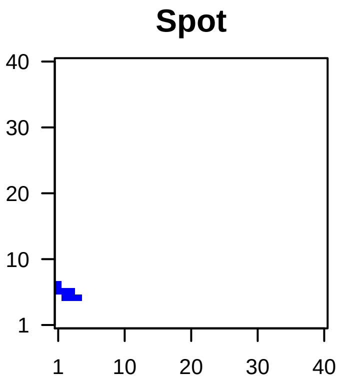

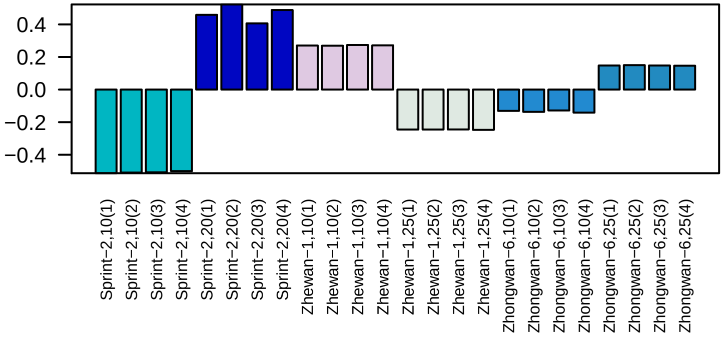

Spot Genelist

Rank	ID	max e	min e	r	Symbol	Description
1	TRINITY_DN4	3.08	−0.97	0.73		
2	TRINITY_DN4	1.2	−0.6	0.76		
3	TRINITY_DN2	1.01	−0.53	0.78		
4	TRINITY_DN4	0.83	−0.44	0.93		
5	TRINITY_DN1	0.79	−0.34	0.73		
6	TRINITY_DN9	0.79	−0.31	0.91		
7	TRINITY_DN2	0.78	−0.52	0.96		
8	TRINITY_DN5	0.78	−0.52	0.92		
9	TRINITY_DN2	0.74	−0.39	0.8		
10	TRINITY_DN5	0.72	−0.42	0.8		
11	TRINITY_DN1	0.71	−0.32	0.88		
12	TRINITY_DN1	0.67	−0.39	0.94		
13	TRINITY_DN1	0.67	−0.67	0.89		
14	TRINITY_DN3	0.65	−0.56	0.87		
15	TRINITY_DN4	0.64	−0.32	0.78		
16	TRINITY_DN4	0.62	−0.68	0.78		
17	TRINITY_DN1	0.62	−0.53	0.93		
18	TRINITY_DN3	0.62	−0.68	0.78		
19	TRINITY_DN3	0.6	−0.42	0.91		
20	TRINITY_DN2	0.6	−0.37	0.86		

K−Means Cluster

Spot Summary: M

metagenes = 7
genes = 40

<r> metagenes = 1
<r> genes = 0.62
beta: r2= 24.62 / log p= −Inf

samples with spot = 11 (45.8 %)
Sprint−2(20) : 3 (75 %)
Zhewan−1(10) : 4 (100 %)
Zhongwan−6(10) : 4 (100 %)

Spot Genelist

Rank	ID	max e	min e	r	Description	Symbol
1	TRINITY_DN2	23.2	−19.57	0.81		
2	TRINITY_DN1	21.33	−106.92	0.92		
3	TRINITY_DN7	20.26	−13.18	0.8		
4	TRINITY_DN4	19.94	−13.52	0.56		
5	TRINITY_DN3	16.43	−9.68	0.66		
6	TRINITY_DN1	13.69	−6.37	0.67		
7	TRINITY_DN7	13.41	−12.69	0.9		
8	TRINITY_DN3	13.38	−29.37	0.9		
9	TRINITY_DN2	11.58	−8.49	0.75		
10	TRINITY_DN3	10.97	−5.32	0.59		
11	TRINITY_DN3	9.96	−9.05	0.54		
12	TRINITY_DN7	7.7	−5.96	0.71		
13	TRINITY_DN3	5.8	−7.86	0.77		
14	TRINITY_DN3	5.67	−6.93	0.8		
15	TRINITY_DN3	5.25	−4.21	0.68		
16	TRINITY_DN3	4.83	−3.23	0.71		
17	TRINITY_DN3	4.13	−3.93	0.79		
18	TRINITY_DN1	4.04	−4.03	0.74		
19	TRINITY_DN6	3.63	−3.54	0.87		
20	TRINITY_DN1	3.44	−2.28	0.77		

Overview Map

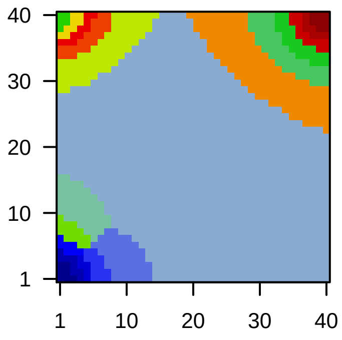

Spot

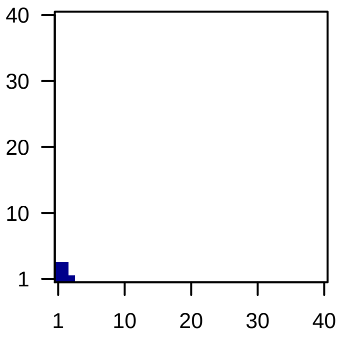

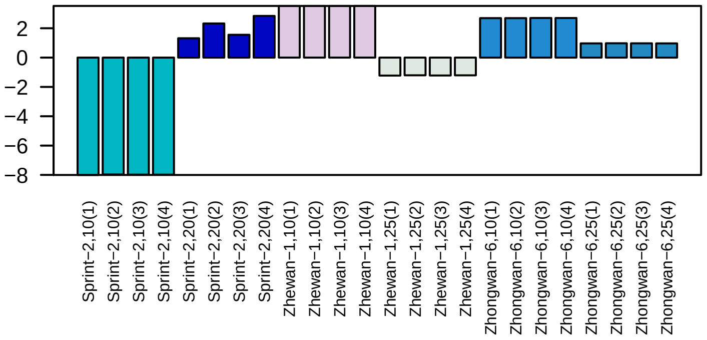

K−Means Cluster

Spot Summary: N

metagenes = 74
genes = 771

<r> metagenes = 0.79
<r> genes = 0.55
beta: r2= 0.03 / log p= −0.35

samples with spot = 0 (0 %)

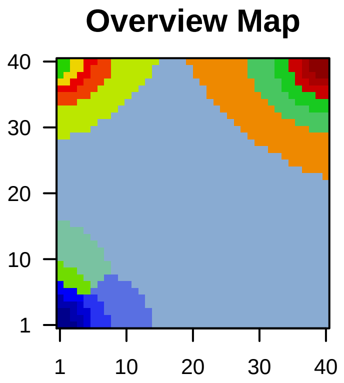

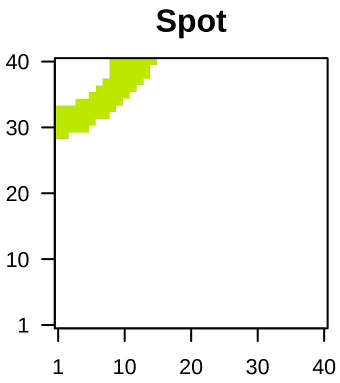

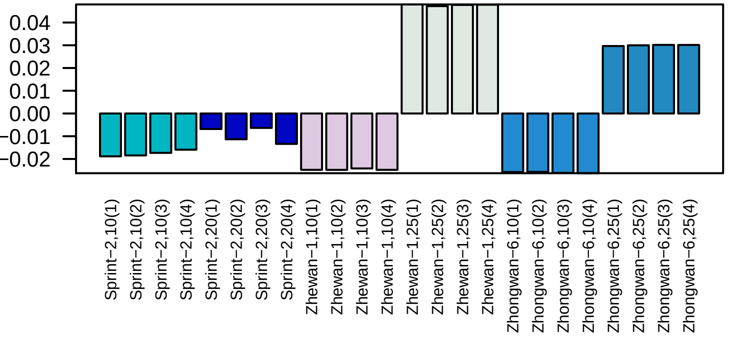

Spot Genelist

Rank	ID	max e	min e	r	Symbol	Description
1	TRINITY_DN5	0.38	−0.19	0.35		
2	TRINITY_DN9	0.34	−0.07	0.48		
3	TRINITY_DN8	0.27	−0.15	0.46		
4	TRINITY_DN3	0.24	−0.15	0.46		
5	TRINITY_DN5	0.23	−0.14	0.46		
6	TRINITY_DN1	0.22	−0.09	0.7		
7	TRINITY_DN5	0.22	−0.15	0.39		
8	TRINITY_DN4	0.21	−0.09	0.7		
9	TRINITY_DN1	0.2	−0.28	0.28		
10	TRINITY_DN1	0.2	−0.12	0.7		
11	TRINITY_DN2	0.19	−0.13	0.51		
12	TRINITY_DN1	0.19	−0.18	0.57		
13	TRINITY_DN3	0.19	−0.22	0.51		
14	TRINITY_DN9	0.19	−0.1	0.44		
15	TRINITY_DN6	0.19	−0.14	0.65		
16	TRINITY_DN3	0.18	−0.09	0.57		
17	TRINITY_DN1	0.18	−0.09	0.57		
18	TRINITY_DN1	0.18	−0.07	0.72		
19	TRINITY_DN2	0.18	−0.04	0.47		
20	TRINITY_DN8	0.17	−0.11	0.48		

K−Means Cluster

Spot Summary: O

metagenes = 8
genes = 28

<r> metagenes = 0.99
<r> genes = 0.7
beta: r2= 2.61 / log p= −Inf

samples with spot = 0 (0 %)

Spot Genelist

Rank	ID	max e	min e	r	Symbol	Description
1	TRINITY_DN1	1.98	−1.06	0.77		
2	TRINITY_DN5	1.61	−0.68	0.87		
3	TRINITY_DN2	1.37	−0.4	0.55		
4	TRINITY_DN3	1.31	−0.35	0.91		
5	TRINITY_DN4	1.27	−0.49	0.87		
6	TRINITY_DN3	1.25	−0.86	0.86		
7	TRINITY_DN8	1.14	−0.66	0.95		
8	TRINITY_DN1	0.97	−0.43	0.68		
9	TRINITY_DN1	0.94	−0.42	0.89		
10	comp13092_c	0.93	−0.67	0.8		
11	TRINITY_DN1	0.87	−0.34	0.87		
12	TRINITY_DN6	0.8	−0.54	0.89		
13	comp27570_c	0.79	−0.42	0.97		
14	TRINITY_DN2	0.76	−0.45	0.98		
15	TRINITY_DN2	0.74	−0.86	0.39		
16	comp22764_c	0.74	−0.75	0.73		
17	TRINITY_DN5	0.73	−0.4	0.99		
18	TRINITY_DN2	0.66	−0.44	0.97		
19	TRINITY_DN1	0.64	−0.36	0.74		
20	TRINITY_DN4	0.64	−0.24	0.97		

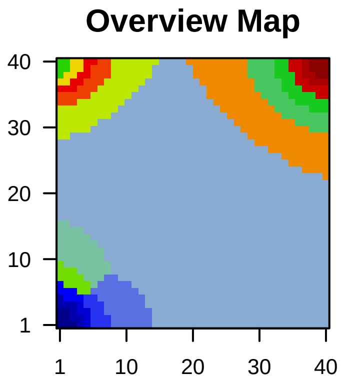

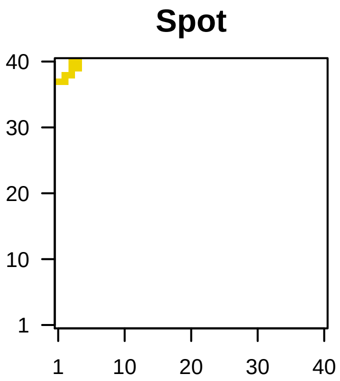

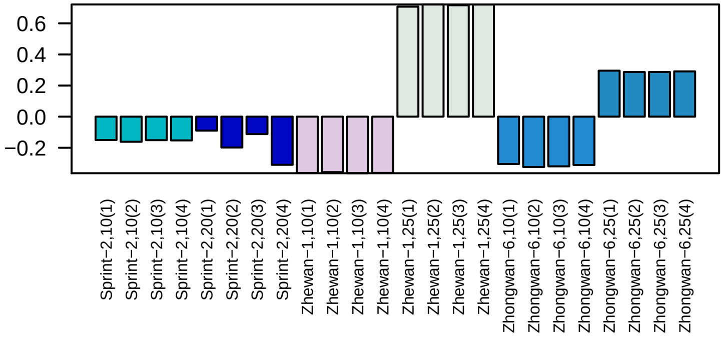

K−Means Cluster

Spot Summary: P

metagenes = 22
genes = 168

<r> metagenes = 0.92
<r> genes = 0.63
beta: r2= 0.24 / log p= −1.83

samples with spot = 0 (0 %)

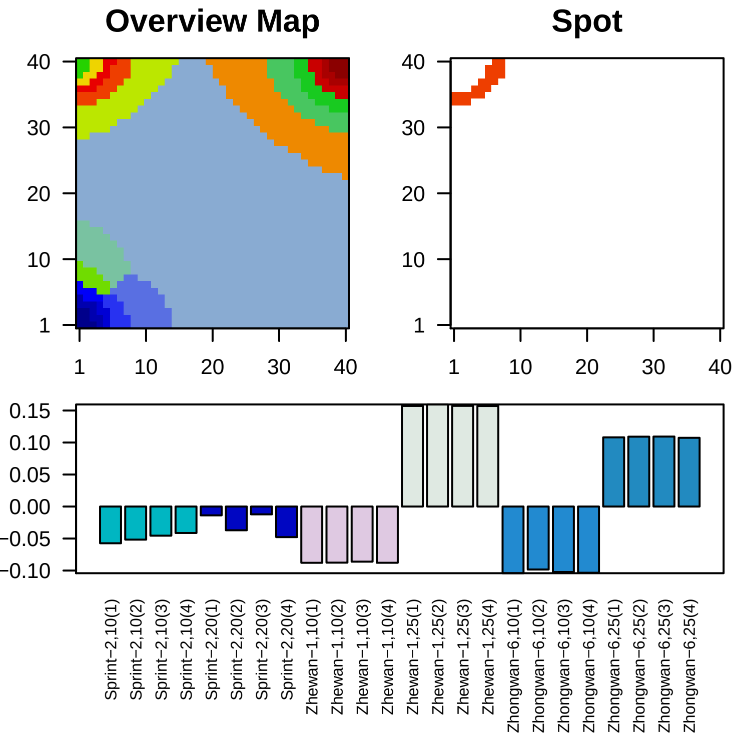

Spot Genelist

Rank	ID	max e	min e	r	Symbol	Description
1	TRINITY_DN1	0.63	−0.43	0.66		
2	comp39331_c	0.55	−0.52	0.49		
3	TRINITY_DN7	0.5	−0.15	0.41		
4	TRINITY_DN5	0.49	−0.23	0.64		
5	TRINITY_DN5	0.45	−0.29	0.62		
6	TRINITY_DN3	0.43	−0.22	0.87		
7	TRINITY_DN9	0.4	−0.11	0.53		
8	TRINITY_DN1	0.38	−0.2	0.44		
9	TRINITY_DN5	0.37	−0.22	0.77		
10	TRINITY_DN1	0.36	−0.19	0.83		
11	TRINITY_DN4	0.36	−0.26	0.73		
12	TRINITY_DN4	0.36	−0.12	0.78		
13	TRINITY_DN9	0.35	−0.26	0.81		
14	TRINITY_DN5	0.35	−0.22	0.53		
15	TRINITY_DN5	0.35	−0.21	0.82		
16	TRINITY_DN2	0.34	−0.22	0.85		
17	TRINITY_DN2	0.34	−0.12	0.81		
18	comp40010_c	0.32	−0.11	0.8		
19	TRINITY_DN3	0.31	−0.22	0.42		
20	comp40760_c	0.31	−0.06	0.72		

K−Means Cluster

Spot Summary: Q

metagenes = 10
genes = 85

<r> metagenes = 0.97
<r> genes = 0.74
beta: r2= 0.85 / log p= −9.74

samples with spot = 0 (0 %)

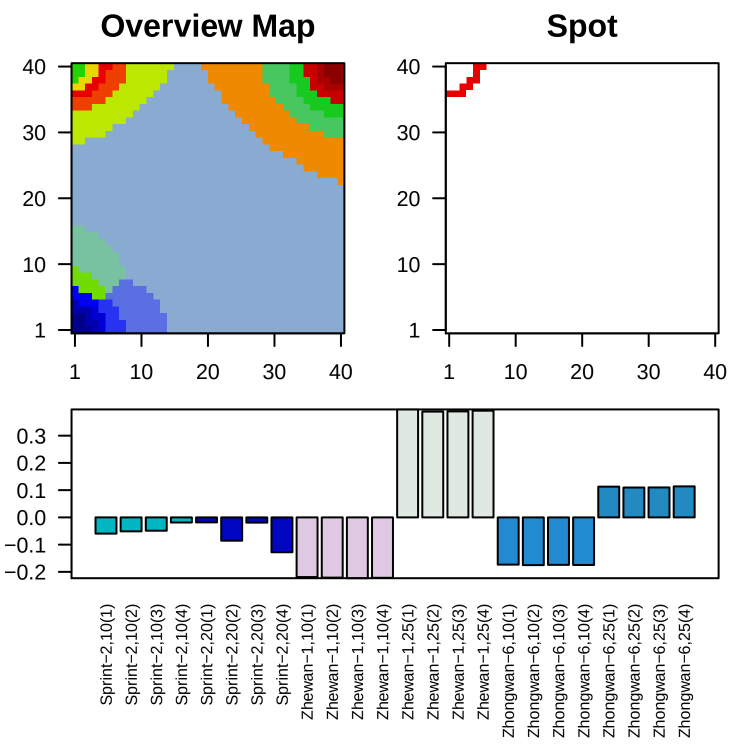

Spot Genelist

Rank	ID	max e	min e	r	Symbol	Description
1	TRINITY_DN1	0.86	−0.48	0.84		
2	TRINITY_DN3	0.86	−0.5	0.35		
3	TRINITY_DN4	0.81	−0.4	0.89		
4	TRINITY_DN1	0.69	−0.3	0.9		
5	TRINITY_DN3	0.68	−0.38	0.9		
6	TRINITY_DN4	0.66	−0.38	0.9		
7	TRINITY_DN7	0.65	−0.54	0.3		
8	TRINITY_DN2	0.62	−0.32	0.74		
9	TRINITY_DN6	0.61	−0.33	0.65		
10	TRINITY_DN8	0.6	−0.35	0.38		
11	TRINITY_DN2	0.59	−0.2	0.79		
12	TRINITY_DN8	0.59	−0.37	0.95		
13	TRINITY_DN4	0.59	−0.19	0.87		
14	TRINITY_DN2	0.58	−0.55	0.89		
15	TRINITY_DN6	0.58	−0.4	0.7		
16	TRINITY_DN1	0.58	−0.2	0.94		
17	TRINITY_DN1	0.57	−0.32	0.91		
18	TRINITY_DN4	0.57	−0.22	0.57		
19	TRINITY_DN1	0.57	−0.43	0.85		
20	TRINITY_DN7	0.55	−0.41	0.86		

K−Means Cluster

Spot Summary: R

metagenes = 5
genes = 21

<r> metagenes = 1
<r> genes = 0.75
beta: r2= 8.95 / log p= −Inf

samples with spot = 9 (37.5 %)
Sprint−2(20) : 1 (25 %)
Zhewan−1(25) : 4 (100 %)
Zhongwan−6(25) : 4 (100 %)

Spot Genelist

Rank	ID	max e	min e	r	Symbol	Description
1	TRINITY_DN2	19.02	−23.73	0.94		
2	TRINITY_DN6	13.45	−12.66	0.97		
3	TRINITY_DN2	10.34	−9.73	0.93		
4	TRINITY_DN4	10.09	−9.97	0.95		
5	TRINITY_DN4	9.16	−4.51	0.88		
6	TRINITY_DN2	8.02	−4.57	0.98		
7	TRINITY_DN2	7.51	−8.63	0.93		
8	TRINITY_DN4	6.31	−3.15	0.94		
9	comp39373_c	6.03	−3.43	0.98		
10	TRINITY_DN2	3.51	−1.01	0.86		
11	TRINITY_DN3	2.68	−3.01	0.62		
12	TRINITY_DN1	2.46	−0.77	0.76		
13	TRINITY_DN3	2.45	−1.45	0.98		
14	TRINITY_DN1	2.42	−1.79	0.85		
15	TRINITY_DN1	2.2	−0.88	0.69		
16	TRINITY_DN3	1.73	−0.78	0.89		
17	TRINITY_DN2	1.69	−1.29	0.89		
18	TRINITY_DN7	1.61	−1.45	0.68		
19	TRINITY_DN1	1.4	−0.89	0.66		
20	TRINITY_DN4	1.08	−0.63	0.8		

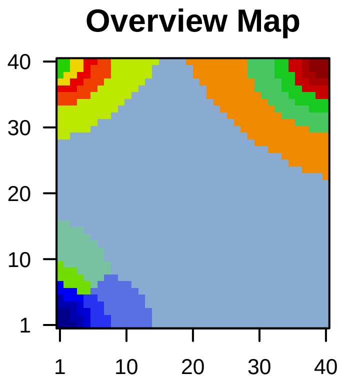

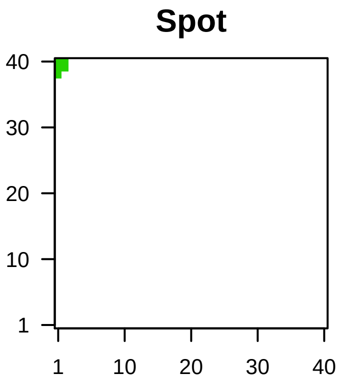

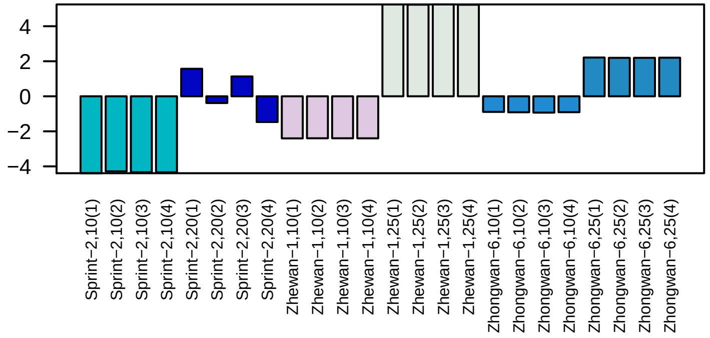

K−Means Cluster

Spot Summary: S

metagenes = 13
genes = 162

<r> metagenes = 0.95
<r> genes = 0.77
beta: r2= 0.75 / log p= −7.36

samples with spot = 0 (0 %)

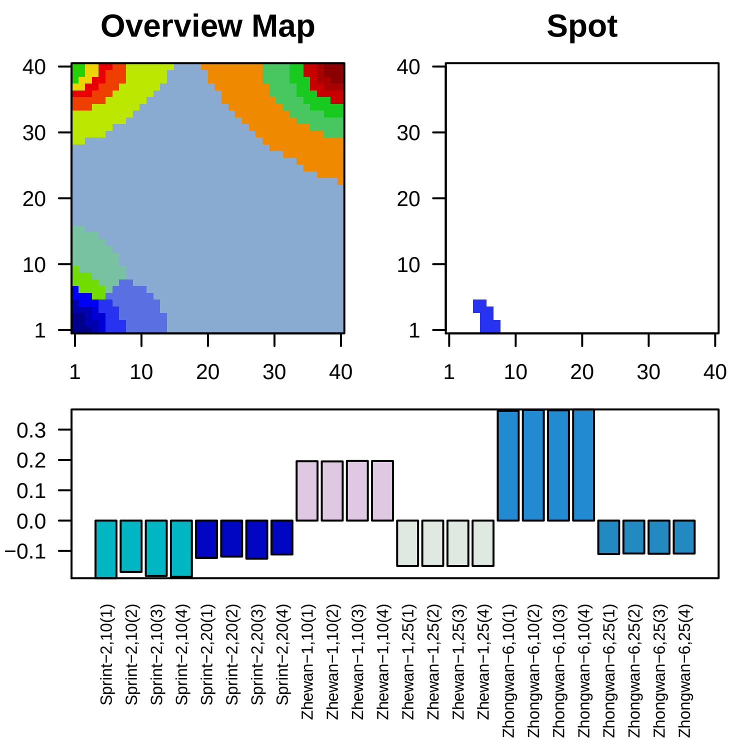

Spot Genelist

Rank	ID	max e	min e	r	Symbol	Description
1	TRINITY_DN4	1.56	−0.78	0.82		
2	TRINITY_DN7	1.25	−0.29	0.83		
3	TRINITY_DN8	1.23	−0.34	0.85		
4	TRINITY_DN2	1.07	−0.26	0.86		
5	TRINITY_DN1	0.95	−0.38	0.9		
6	TRINITY_DN1	0.94	−0.2	0.87		
7	TRINITY_DN1	0.92	−0.25	0.6		
8	TRINITY_DN4	0.89	−0.45	0.96		
9	TRINITY_DN4	0.88	−0.2	0.8		
10	TRINITY_DN2	0.88	−0.32	0.97		
11	comp36789_c	0.86	−0.48	0.91		
12	TRINITY_DN1	0.84	−0.22	0.55		
13	TRINITY_DN4	0.83	−0.21	0.91		
14	TRINITY_DN3	0.83	−0.45	0.68		
15	TRINITY_DN5	0.82	−0.19	0.86		
16	TRINITY_DN4	0.81	−0.2	0.88		
17	TRINITY_DN1	0.8	−0.27	0.92		
18	TRINITY_DN4	0.8	−0.39	0.6		
19	TRINITY_DN3	0.78	−0.16	0.8		
20	TRINITY_DN6	0.78	−0.3	0.66		

K−Means Cluster

Spot Summary: T

metagenes = 46
genes = 693

<r> metagenes = 0.88
<r> genes = 0.68
beta: r2= 0.08 / log p= −0.74

samples with spot = 0 (0 %)

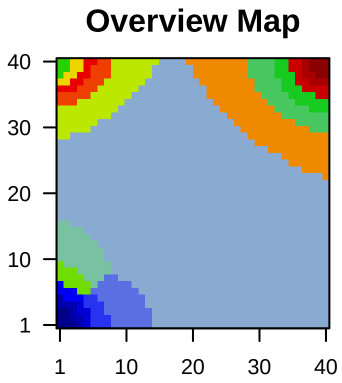

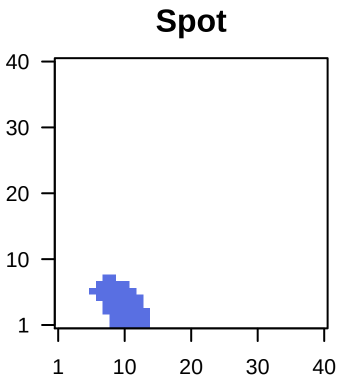

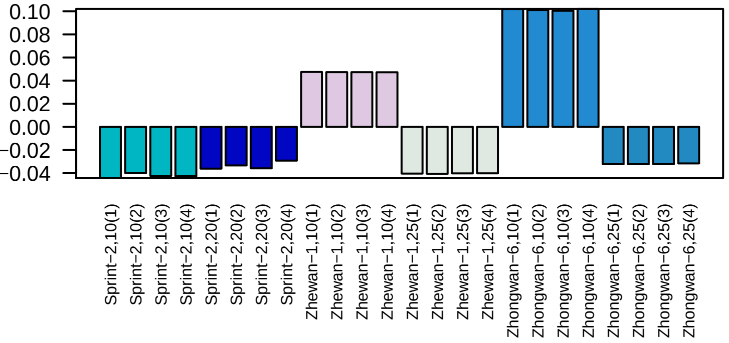

Spot Genelist

Rank	ID	max e	min e	r	Symbol	Description
1	TRINITY_DN5	0.52	−0.24	0.72		
2	TRINITY_DN6	0.5	−0.1	0.71		
3	TRINITY_DN7	0.43	−0.35	0.58		
4	TRINITY_DN3	0.42	−0.08	0.87		
5	TRINITY_DN1	0.4	−0.37	0.62		
6	TRINITY_DN1	0.39	−0.14	0.61		
7	TRINITY_DN5	0.39	−0.07	0.85		
8	TRINITY_DN4	0.39	−0.09	0.87		
9	TRINITY_DN4	0.38	−0.13	0.22		
10	TRINITY_DN2	0.37	−0.11	0.76		
11	TRINITY_DN2	0.36	−0.21	0.6		
12	TRINITY_DN5	0.36	−0.06	0.84		
13	TRINITY_DN1	0.34	−0.12	0.81		
14	TRINITY_DN1	0.34	−0.08	0.54		
15	comp23505_c	0.34	−0.09	0.95		
16	TRINITY_DN2	0.33	−0.09	0.9		
17	TRINITY_DN2	0.33	−0.06	0.83		
18	TRINITY_DN1	0.33	−0.06	0.84		
19	TRINITY_DN7	0.33	−0.14	0.85		
20	TRINITY_DN2	0.33	−0.07	0.88		
